# Is exclusion of leukocytes from platelet-rich plasma (PRP) a better choice for early intervertebral disc regeneration?

**DOI:** 10.1186/s13287-018-0937-7

**Published:** 2018-07-18

**Authors:** Shan-zheng Wang, Wei-min Fan, Jun Jia, Liang-yu Ma, Jia-bin Yu, Chen Wang

**Affiliations:** 1 The First Clinical School of Nanjing Medical University, 300 Guangzhou Road, Nanjing, Jiangsu 210029 People’s Republic of China; 2 Department of Orthopaedics, Zhongda Hospital, Medical School of Southeast University, 87 Ding Jia Qiao Road, Nanjing, Jiangsu 210009 People’s Republic of China

**Keywords:** Platelet-rich plasma, Leukocyte- and platelet-rich plasma, Pure platelet-rich plasma, Intervertebral disc degeneration, Nucleus pulposus, Stem cells

## Abstract

**Background:**

Platelet-rich plasma (PRP) is becoming a promising strategy to treat early intervertebral disc degeneration (IDD) in clinics. Pure PRP without leukocytes (P-PRP) may decrease the catabolic and inflammatory changes in the early degenerated intervertebral discs. The aim of this study was to investigate the effects of P-PRP on nucleus pulposus-derived stem cells (NPSCs) isolated from early degenerated intervertebral discs in vitro.

**Methods:**

NPSCs isolated from early degenerated discs of rabbits were treated with P-PRP or leukocyte-platelet-rich PRP (L-PRP) in vitro, followed by measuring cell proliferation, stem cell marker expression, inflammatory gene expression, and anabolic and catabolic protein expression by immunostaining, quantitative real-time polymerase chain reaction, Western blot, and enzyme-linked immunosorbent assay.

**Results:**

Cell proliferation was induced by P-PRP in a dose-dependent manner with maximum proliferation at 10% P-PRP dose. P-PRP induced differentiation of NPSCs into active nucleus pulposus cells. P-PRP mainly increased the expression of anabolic genes and relative proteins, aggrecan (AGC), collagen types II (Col II), while L-PRP predominantly increased the expression of catabolic and inflammatory genes, matrix metalloproteinase-1 (MMP-1), MMP-13, interleukin-1 beta (IL-1β), IL-6, tumor necrosis factor alpha (TNF-α), and protein production of IL-1β and TNF-α.

**Conclusions:**

Leukocytes in PRP activate inflammatory and catabolic effects on NPSCs from early degenerated intervertebral discs. Hence, P-PRP may be a more suitable therapeutic strategy for early IDD.

## Background

As a major cause of low back pain, intervertebral disc degeneration (IDD) is a serious clinical problem, causing enormous financial and health-care costs [1, 2]. Conservative treatments, including nonsteroidal anti-inflammatory drugs and physiotherapy, can often alleviate the symptoms but without prohibiting the degenerative process. Spinal surgeries are extensively accepted as an effective strategy to treat IDD with worsening neurological symptoms [3, 4]. However, after removing the herniated discs or even fusing the adjacent spinal segments, the neighboring discs may experience an increasing progression of degeneration and instability [5]. In recent years, as a choice of biological strategies, platelet-rich plasma (PRP) is drawing increasing attention both in clinical treatment and basic scientific research [6–8].

PRP is defined as a volume of the autologous plasma with high platelet concentration above baseline [9, 10]. The concept that PRP treatment can reverse the progression of IDD is based on the role of platelets in wound-healing [10–14]. When activated, a variety of growth factors, including platelet-derived growth factor (PDGF), transforming growth factor-β (TGF-β), vascular endothelial growth factor-A (VEGF-A), basic fibroblast growth factor (bFGF), epidermal growth factor (EGF), and connective tissue growth factor (CTGF), among others, are secreted from platelets [15, 16]. All these growth factors play a joint role when applied in intervertebral disc regeneration. Despite the wide use of PRP, there are no standardized protocols in PRP preparation, resulting in variations in componential composition, in particular, leukocytes [17]. It has been demonstrated that leukocytes in PRP can release high levels of pro-inflammatory cytokines, such as IL-1β and TNF-α, which increase the catabolism of extracellular matrix (ECM) [18, 19]. However, leukocytes in PRP can be beneficial to stimulate the immune response against infection and promote chemotaxis, proliferation, and differentiation of cells [20]. Therefore, it is necessary to define the precise effects of PRP on degenerated discs so that its clinical applications can be more accurate and beneficial.

Leukocyte-platelet-rich PRP (L-PRP) is often used to treat open traumatic injuries [21]. However, high levels of pro-inflammatory cytokines in L-PRP may activate the catabolic and inflammatory changes to counter the beneficial effect of the growth factors on tissue regeneration. Considering the detrimental effects of leukocytes on tissue regeneration, to exclude the leukocytes in PRP for pure PRP (P-PRP) is an attempt to achieve maximum therapeutic potential.

As a core component of intervertebral disc, nucleus pulposus (NP) is playing an important role in the mechanical and biological homeostasis of intervertebral discs [22]. In recent years, NP -derived stem cells (NPSCs) were isolated and characterized [23, 24]. As a key role in intervertebral disc homeostasis, these endogenic stem cells self-renew and differentiate into NP cells that are responsible for the self-regeneration of the NP when activated. While the superior effects of P-PRP over L-PRP have been proved on tendon [25], bone [26] and cartilage [27] healing, they have not yet been elucidated on intervertebral disc regeneration. Also, how P-PRP differentiate from L-PRP in influencing the endogenic NPSCs when repairing the early degenerated intervertebral discs is largely unknown. Therefore, we designed this study to determine effects of P-PRP on NPSCs isolated from early degenerated intervertebral discs.

## Methods

### Preparation and quantification of P-PRP and L-PRP

The Institutional Animal Care and Use Committee of Southeast University approved the protocol for the use of rabbits in this study. In total, 20 adult New Zealand White rabbits (6–8 months old, 3.0–4.0 kg) were used in this study, and were randomly used in either P-PRP group or L-PRP group (*n* = 10 each). P-PRP and L-PRP were prepared according to a two-step centrifugation method as previously described [27]. Briefly, the fresh whole blood was collected into acid citrate dextrose solution A (ACD-A) anticoagulant (1 mL ACD-A/9 mL blood) and centrifuged at 160 g for 10 min. After the first centrifugation, the blood was separated into three layers: erythrocytes at the bottom, buffy coat in the middle, and platelet-containing plasma at the top. Then, the top layer was transferred to a new tube and spun again at 1000 g for 10 min. After discarding the supernatant (platelet-poor plasma), the remaining plasma and precipitated platelets were blended evenly to obtain 1 mL of P-PRP. L-PRP was prepared based using a buffy coat two-step centrifugation method. In brief, a centrifugation at 250 g for 10 min was used to separate three layers as above. Then, the top two layers were collected and further centrifuged at 1000 g for 10 min. After the second centrifugation, the supernatant (platelet-poor plasma) was discarded, and the precipitated platelets were resuspended in the remaining 1 mL of plasma to obtain L-PRP. Leukocyte and platelet concentrations in L-PRP, P-PRP, and whole blood were measured by an automatic hematology analyzer (XE-2100, Sysmex, Kobe, Japan).

### Isolation of rabbit NPSCs from early degenerated and healthy discs

The rabbit lumbar IDD models were established as previously described [28]. After 2 weeks of intervention, the early degenerated disc model was evaluated by a 3.0 T magnetic resonance imaging (MRI). The NP tissues of the early degenerated discs and healthy discs (control group) were isolated and cultured. Briefly, the harvested NP tissues were minced and digested using 0.2 mg/ml collagenase II (Sigma-Aldrich, St. Louis, Mo, USA) in Dulbecco’s modified Eagle’s medium with low glucose (DMEM-LG, Gibco, Waltham, MA, USA) for 4–6 h. The suspension was then centrifuged at 1000 rpm for 10 min. The cell pellet was re-suspended in DMEM-LG supplemented with 10% FBS, 100 U/mL penicillin, 100 mg/mL streptomycin. The cells were diluted to 10 cells/uL and seeded into six-well plates in a humidified incubator at 37 °C with 5% CO_2_. After 15 days, NPSCs colonies observed in the wells were trypsinized for further culture. NPSCs at passage 2 were used for further experiments. To compare the formed colonies, the initially plated cells from NP tissues were cultured for 10 days, washed three times by PBS, fixed with 10% methanol for 15 min and stained with 0.5% crystal violet (Sigma-Aldrich) for 15 min for further observation.

### Polymerase chain reaction (PCR) for stem cell markers

Total RNA was extracted from cells and then reverse transcribed to cDNA using a reverse transcription kit (Fermentas, Waltham, MA, USA) after DNase I treatment (Fermentas). The genes included MSC positive marker genes (CD29, CD44, CD166), MSC negative marker genes (CD4, CD8, CD14), and housekeeping gene GAPDH [29]. All the primer sequences are listed in Table 1. The sequences were designed according to published papers or designed by ourselves. All the primers were synthesized by Invitrogen.

**Table 1:** Primers used in polymerase chain reaction and quantitative real-time polymerase chain reaction for gene expression analysis

Gene	Primer sequence (5′to3′)
CD29-F	GTCACCAACCGTAGCAA
CD29-R	CTCCTCATCTCATTCATCAG
CD44-F	CGATTTGAATATAACCTGCCGC
CD44-R	CGTGCCCTTCTATGAACCCA
CD166-F	GGACAGCCCGAAGGAATACGAA
CD166-R	GACACAGGCAGGGAATCACCAA
CD4-F	GATGGAGGTGGAACTGC
CD4-R	GGAAAGCCCAACACTATG
CD8-F	GGGTGGAAAAGGAGAAGC
CD8-R	AGGTGAGTGCGGGAGAC
CD14-F	CAGGTGCCTAAGGGACT
CD14-R	AATAAAGTGGGAAGCGG
Coll II-F	CAGGATGTCCAGGAGGCT
Coll II-R	GCAGTGGCGAGGTCAGTAG
AGC-F	GGAGCCCGAGCCTATACTATTT
AGC-R	CCCAAGGACCACCAATCA
IL-6-F	AGGGAGGTCGAGCTGTTCTC
IL-6-R	GGAGTGTTCACTAAGCGGTCA
IL-1β-F	CGGTCAAGGAGAGGAGCTTAC
IL-1β-R	GGACTAGCCCTCGCTTATCTTT
TNF-α-F	GGAGAAGCCGGTAGTGGAGAT
TNF-α-R	GGTCTGGTCACGGTTTGGAA
IL-8-F	CACAGTGGACGACATCCGAAA
IL-8-R	AGCTACATAGGAATTACGGGCAA
MMP-1-F	CGACTCGCTATCTCCAAGTGA
MMP-1-R	GTTGAACCAGTCTCCGACCA
MMP-13-F	GGAGGCGAGAACATCAAGCC
MMP-13-R	CGGCCTTCCCTCGTAGTGA
OCT4-F	ACCTTCATCGGAAACTCCAAAG
OCT4-R	ACTGTTAGGCTCAGGTGAACT
Nanog-F	CTGTGGGTTTCTGTGCTGG
Nanog-R	CCGGCTTCAAGGCTTTCAG
GAPDH-F	ACTTTGTGAAGCTCATTTCCTGGTA
GAPDH-R	GTGGTTTGAGGGCTCTTACTCCTT

### Induced differentiation potential of isolated NPSCs

The induced differentiation methods were described previously [30, 31]. Briefly, passage 2 NPSCs were seeded at a density of 4 × 10^4^ cells/well in a 24-well plate in basic culture medium (DMEM-LG) supplemented with 10% FBS, 100 U/ml penicillin, 100 mg/ml streptomycin. They were subjected to induced differentiation by culturing them in osteogenic (Cyagen Biosciences, Santa Clara, CA, USA, RASMX-90021), adipogenic (Cyagen Biosciences, RASMX-90031) and chondrogenic (Cyagen Biosciences, RASMX-90041) medium, respectively. The outcomes were evaluated using Alizarin Red, Oil Red O and Alcian Blue staining, respectively.

### NPSCs proliferation assay

NPSCs were seeded at the density of 1 × 10^4^ in a 24-transwell system and cultured in DMEM-LG containing P-PRP or L-PRP at different concentrations: 0%, 5%, 10%, 15%, and 20% (vol/vol). Cell growth was tested on day 3 and 7 by Cell Counting Kit-8 (CCK-8) (Sigma-Aldrich). Fresh culture medium containing 10% CCK-8 solution was added and incubated at 37 °C for 2.5 h. Then, the absorbance was measured by a microplate reader (SPECTRA max Plus 384, Molecular Devices, San Jose, CA, USA) at 450 nm. Each treatment was replicated three times and the absorption values were independently calculated as OD 450nm_experiment_ - OD 450nm_blank_. The average absorption value of all replicates represented cell proliferation in each group.

### Induced cell morphology

NPSCs were seeded in a 24-transwell system at a density of 1 × 10^4^ per well and incubated in growth medium (DMEM-LG + 10% FBS) alone (control group) or growth medium with 10% P-PRP (P-PRP group) or 10% L-PRP (L-PRP group). The culture medium was changed every 3 days. After 14 days, cell morphology was observed under the inverted microscope.

### Western blot analysis

The Western blot analysis was described previously [30, 31]. The cell culture medium was changed every 3 days in three groups as stated above. After 14 days, NPSCs were harvested and centrifuged to obtain cells in each group. Total proteins were then isolated by a mammalian protein extraction reagent (M-PER) (Thermo Fisher Scientific, Waltham, MA, USA, catalog no.78505) containing 1.5% protease inhibitors (Sigma-Aldrich) and then centrifuged at 14,000 g for 10 min. The supernatant was stored at 4 °C. Protein concentration of the supernatant was evaluated by the BCA protein Assay Reagent Kit (Pierce, Waltham, MA, USA). Equal amounts of total protein from each group were then separated on 12% SDS-PAGE gels (Thermo Fisher Scientific) at a constant 100 V for 60 min. Proteins were transferred to a PVDF membrane (EMD Millipore, Burlington, MA, USA) at 100 V for 30 min. The membrane was then blocked in 5% dry milk/TBS-Tween 20 for 1 h at room temperature and then incubated overnight with anti-collagen type II (Col II) antibody (arigo Bio, Hsinchu City, Taiwan, ROC), anti-Agrecan antibody (Novus Biologicals, Littleton, CO, USA) at a dilution of 1:1000. The membranes were washed in PBS/Tween-20 three times for 10 min each and further incubated with peroxidase-conjugated goat anti-mouse antibody (Santa Cruz Biotechnology, Dallas, TX, USA) at a dilution of 1:5000 in 1% dry milk/PBS for 1 h at room temperature. Finally, the protein bands were detected by an enhanced chemiluminescence (ECL) detection kit (Amersham Biosciences, Piscataway, NJ, USA), followed by exposing the membrane to X-ray films for visualization. Mouse antihuman GAPDH (Chemicon, Temecula, CA, USA) was used as an internal control to verify the loading of equal amounts of proteins in each well.

### Gene expression analysis using quantitative real-time polymerase chain reaction (qRT-PCR)

To determine the effects of P-PRP and L-PRP on the gene expression of NPSCs, quantitative real-time polymerase chain reaction (qRT-PCR) was used to analyze the following genes as previously described [30, 31]: stem cell-related gene (Oct-4, Nango), NP-related genes (Col II and AGC), catabolic genes (MMP-1, MMP-13), and inflammatory marker genes (IL-1β, IL-6, IL-8, and TNF-α). The sequences of primers used in the reactions are listed in Table 1. Each reaction had at least three replicates, and the relative expression of each target gene was calculated by using the 2^−ΔΔCT^ formula.

### Measuring of IL-1β and TNF-α

First, NPSCs were cultured as above either in growth medium alone (control) or in growth medium containing 10% P-PRP or 10% L-PRP. At least three replicates were maintained for each group. After 4 days, cells were obtained by trypsin. The cell pellet was used to estimate the cell count by an auto cellometer (Cellometer Auto T4; Nexcelom Bioscience LLC, Lawrence, MA, USA), and the supernatant was used to measure interleukin (IL)-1β and tumor necrosis factor (TNF)-α levels by a specific enzyme-linked immunosorbent assay (ELISA) kits according to the instructions of the manufacturer (Shanghai Westang Biotechnology, Shanghai, China).

### Immunostaining of induced NPSCs

NPSCs cultured as above for 14 days were collected by trypsinization and fixed for 20 min in 4% paraformaldehyde/PBS. The collected cells were washed with 0.1% Triton-X100/PBS for 5 min. Immunostaining for NP-related proteins, aggrecan and, Col II was performed by blocking the fixed cells in 2% mouse serum. Cells were then incubated with mouse monoclonal anti-AGC antibody (Novus Biologicals), anti-collagen II antibody (arigo Bio) overnight. After washing in PBS three times for 5 min each, the cells were further incubated with Cy3-conjugated goat anti-mouse IgG secondary antibody (1:500; EMD Millipore; catalog no. AP124X4-K) for 1.5 h. After the final wash in PBS, the stained cells were examined by an inverted fluorescence microscope (Olympus, Tokyo, Japan, IX51).

### Statistical analysis

Results are presented as mean ± standard deviation (SD) with no less than three replicates for each experimental condition. Statistical analyses were performed using one-way analysis of variance (ANOVA) followed by Fisher’s protected least significant difference for multiple comparisons. For statistical analysis of other results, two-tailed, paired, or unpaired Student’s *t* test was performed. Differences were considered significant when *P* values were below 0.05.

## Results

### Characterization of L-PRP and P-PRP

**Fig. 1:**
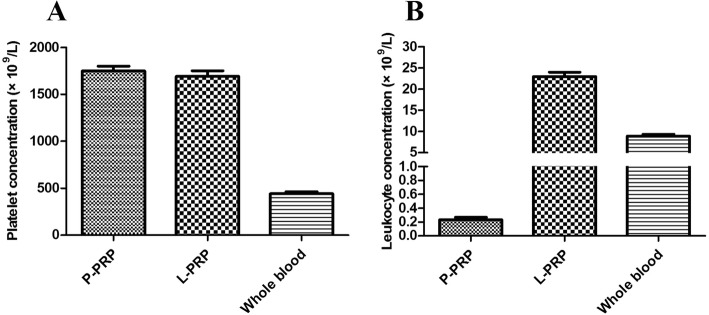
Composition of P-PRP, L-PRP and whole blood. **a** Platelet concentration (× 10^9^/L). **b** Leukocyte concentration (× 10^9^/L). P-PRP pure platelet-rich plasma, L-PRP leukocyte-platelet-rich plasma

Similar platelet concentrations were observed in L-PRP (1691.75 ± 151.89 × 10^9^/L) and P-PRP (1749.13 ± 128.35 × 10^9^/L), which were three times higher than the basic platelet level in the whole blood (440.50 ± 60.18 × 10^9^/L) (Fig. 1a). The average leukocyte concentration in the whole blood was 8.92 ± 1.03 × 10^9^/L. However, in L-PRP, leukocyte concentration was 22.97 ± 2.63 × 10^9^/L, while this concentration was negligible in P-PRP (0.23 ± 0.09 × 10^9^/L) (Fig. 1b).

### Isolation and culture of NPSCs

**Fig. 2:**
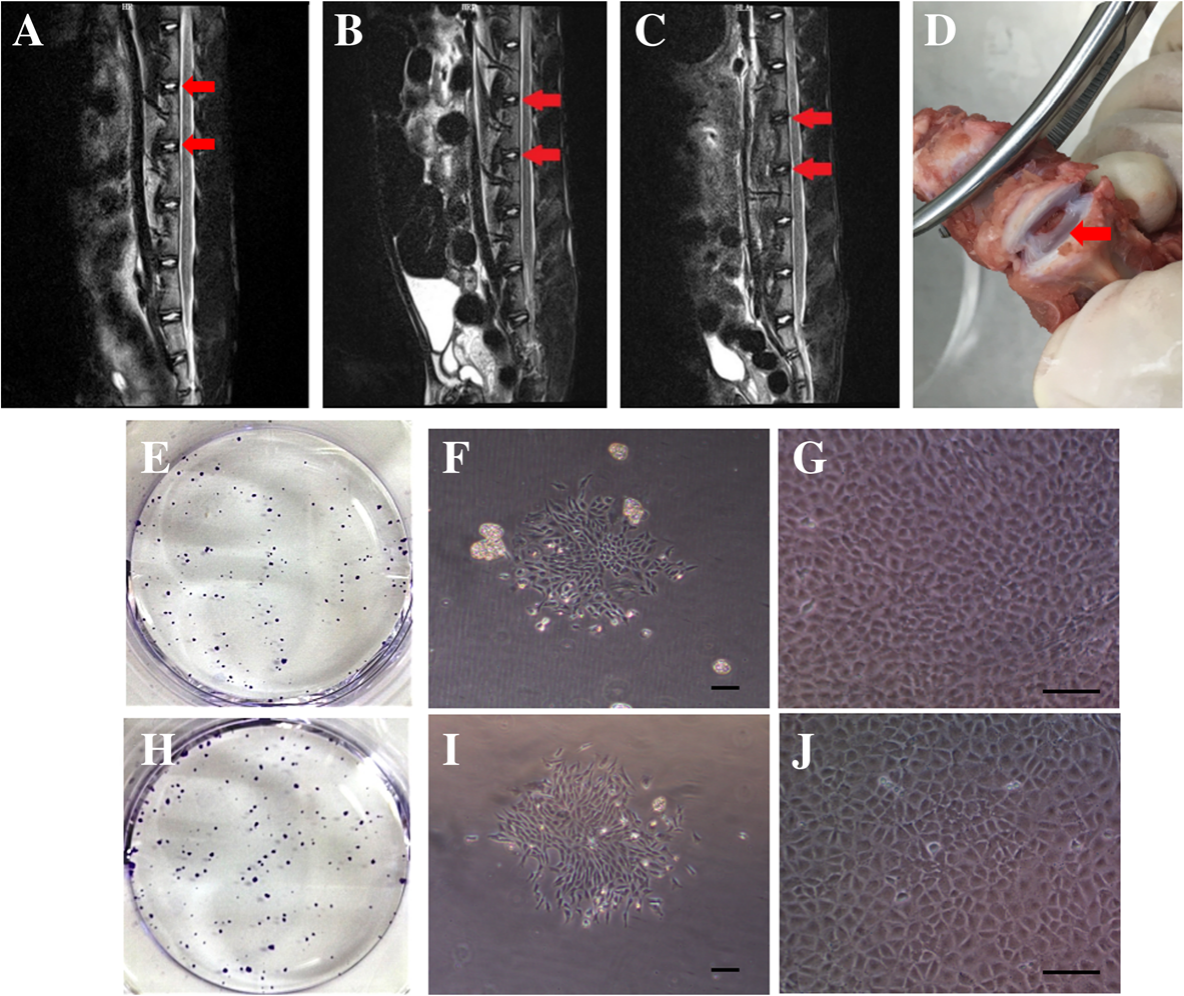
Isolation and culture of NPSCs. The MRI T2-weighted signal of targeted discs (*red arrows*) was in high intensity in the heathy rabbit (**a**). The punctured discs (*red arrows*) showed decreasing signal intensity at week 2 (**b**), and this signal intensity worsened gradually at week 4 (**c**). The degenerated discs were obtained two weeks after intervention, and the NP tissue (*red arrow*) could be seen in the middle of the discs (**d**). NPSCs isolated from early degenerated discs formed colonies after in vitro culture for 10 days as indicated by crystal violet staining (**e**). The morphology of cells in the colonies varied, with some of them being cobblestone-like and others being spindle-like (**f**). As a control, cells isolated from healthy rabbit discs formed more colonies (**h**), but shared similar morphology (**i**). After 2 passages, the morphology of the isolated cells from these two groups all became cobblestone-like (**g**, **j**). Scale bars, 100 mm. NPSCs nucleus pulposus-derived stem cells, MRI magnetic resonance imaging, NP nucleus pulposus

Compared to the healthy discs (Fig. 2a), the punctured discs exhibited decreasing signal intensity after 2 weeks of intervention (Fig. 2b). The worsening degenerative trend was observed at week 4 (Fig. 2c). The NP tissues (Fig. 2d) were collected from the early degenerated discs (2 weeks after intervention). The NP-derived cells isolated from early degenerated discs formed colonies after 10 days as indicated by crystal violet staining (Fig. 2e). The morphology of cells in the colonies also varied, with some of them being cobblestone-like and others being spindle-like (Fig. 2f). At P2, a homogeneous population of cobblestone-like cells was observed (Fig. 2g). As a control, NPSCs isolated from healthy rabbit discs formed more colonies (Fig. 2h), but shared similar morphology (Fig. 2i, j).

### Expression of stem cell markers in NP-derived colony-forming cells

**Fig. 3:**
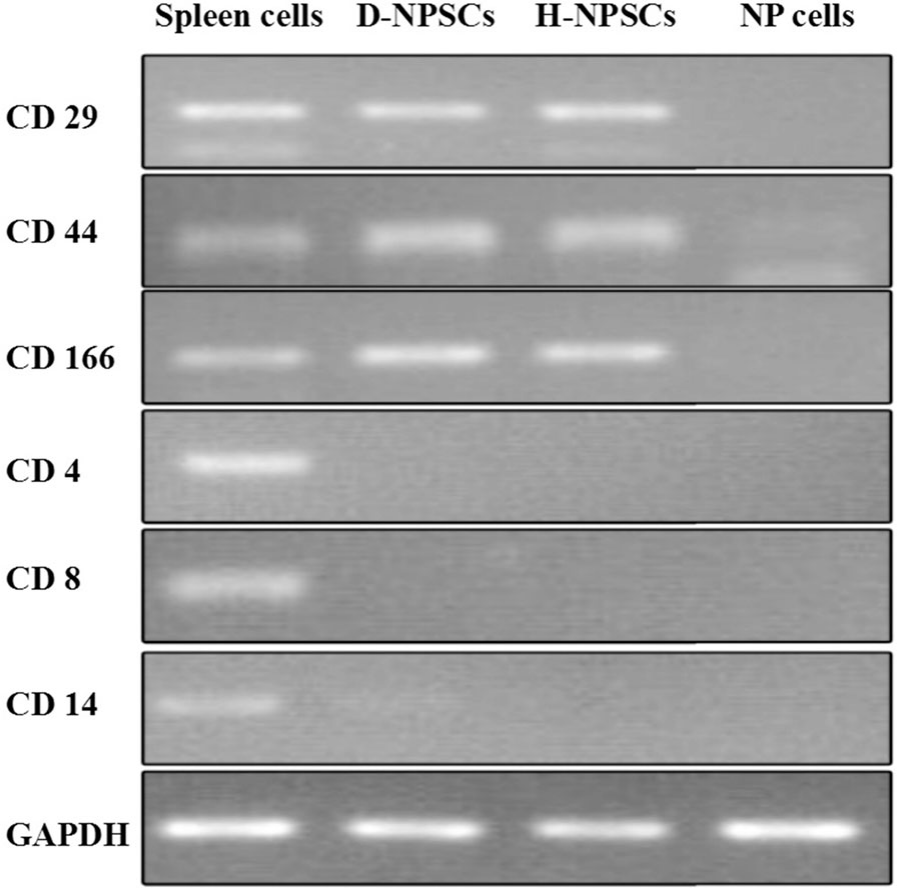
Expression of stem cell markers in NP-derived colony-forming cells. NP-derived colony-forming cells both from the early degenerated discs (D-NPSCs) and healthy discs (H-NPSCs) were positive for CD29, CD44, and CD166, but negative for CD4, CD8 and CD14. Spleen cells expressed all these genes as a positive control, while NP cells expressed none of these genes. *NP* nucleus pulposus

The gene expression of typical MSC-associated surface antigens was tested by PCR (Fig. 3). The NP-derived colony-forming cells isolated from early degenerated discs (D-NPSCs) had strong expression of CD29, CD44, and CD166, which are usually positive in MSCs. Meanwhile, they had negligible expression of CD4, CD8, and CD14, which seldom exist in MSCs. The expression of MSC markers was similarly positive in NPSCs isolated from healthy rabbit discs (H-NPSCs). As a positive control, rabbit spleen cells were found to express all the above genes. As a negative control, NP cells showed negligible expression of all the above genes.

### Multi-potential differentiation of NPSCs

**Fig. 4:**
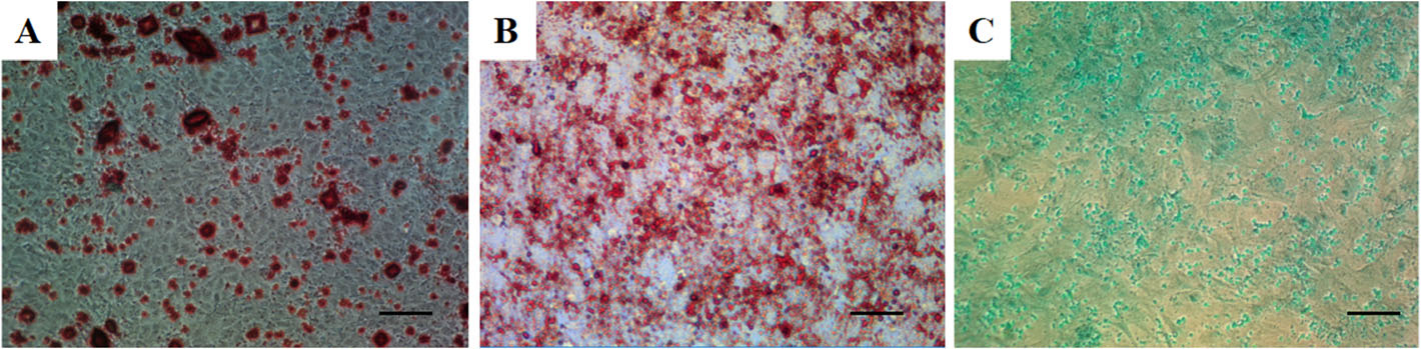
Multi-potential differentiation of NPSCs. Osteogenic differentiation at 3 weeks. Mineralization was positive for Alizarin Red staining (**a**). Adipogenic differentiation at 2 weeks. Secretion of oil droplets were positive for Oil Red O staining (**b**). Chondrogenic differentiation at 3 weeks. Cells were stained with Alcian Blue staining (**c**). Scale bars, 100 mm. *L-PRP* leukocyte-platelet-rich plasma, *NPSCs* nucleus pulposus-derived stem cells, *P-PRP* pure platelet-rich plasma

The rabbit NP-derived colony-forming cells from the early degenerated discs were subjected to induced differentiation processes (osteogenesis, adipogenesis, and chondrogenesis) to determine the multi-differentiation potential. After 3 weeks for osteogenic induction, calcium deposits were highly visible in the induced cells, which were fixed and stained with Alizarin Red (Fig. 4a). During adipogenic induction, the rabbit NPSCs started to secrete oil droplets, which were fixed and stained with Oil Red O staining (Fig. 4b). After 3 weeks for chondrogenic induction, active production of sulfated proteoglycans in the induced cells was validated by Alcian Blue staining (Fig. 4c).

### NPSCs proliferation rate is PRP dose-dependent

**Fig. 5:**
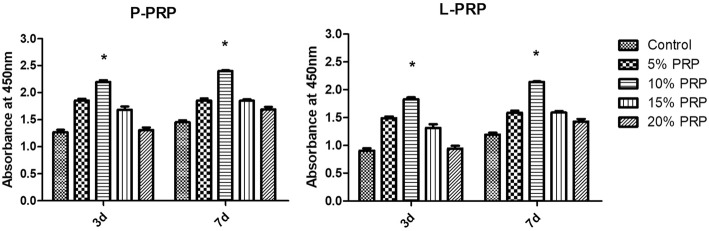
Proliferation of NPSCs cultured in various concentrations of P-PRP or L-PRP. Cell proliferation was measured on day 3 and 7 in culture. Cells proliferated in a dose-dependent manner with 10% P-PRP and 10% L-PRP inducing the maximum effects at each time point. *Asterisks* indicate significant differences (*P* < 0.05) when compared with the controls. *L-PRP* leukocyte-platelet-rich plasma, *NPSCs* nucleus pulposus-derived stem cells, *P-PRP* pure platelet-rich plasma

In the presence of P-PRP or L-PRP, cell proliferation rate increased in a PRP dose-dependent manner (Fig. 5). However, increasing PRP dose over 15% descended the proliferation trend. At each time point, 10% PRP concentration induced significantly higher cell proliferation rate compared with other groups. Since maximum proliferation rate was induced by 10% PRP, this dose was used for further analyses.

### P-PRP and L-PRP specifically induces NPSCs into active NP cells

**Fig. 6:**
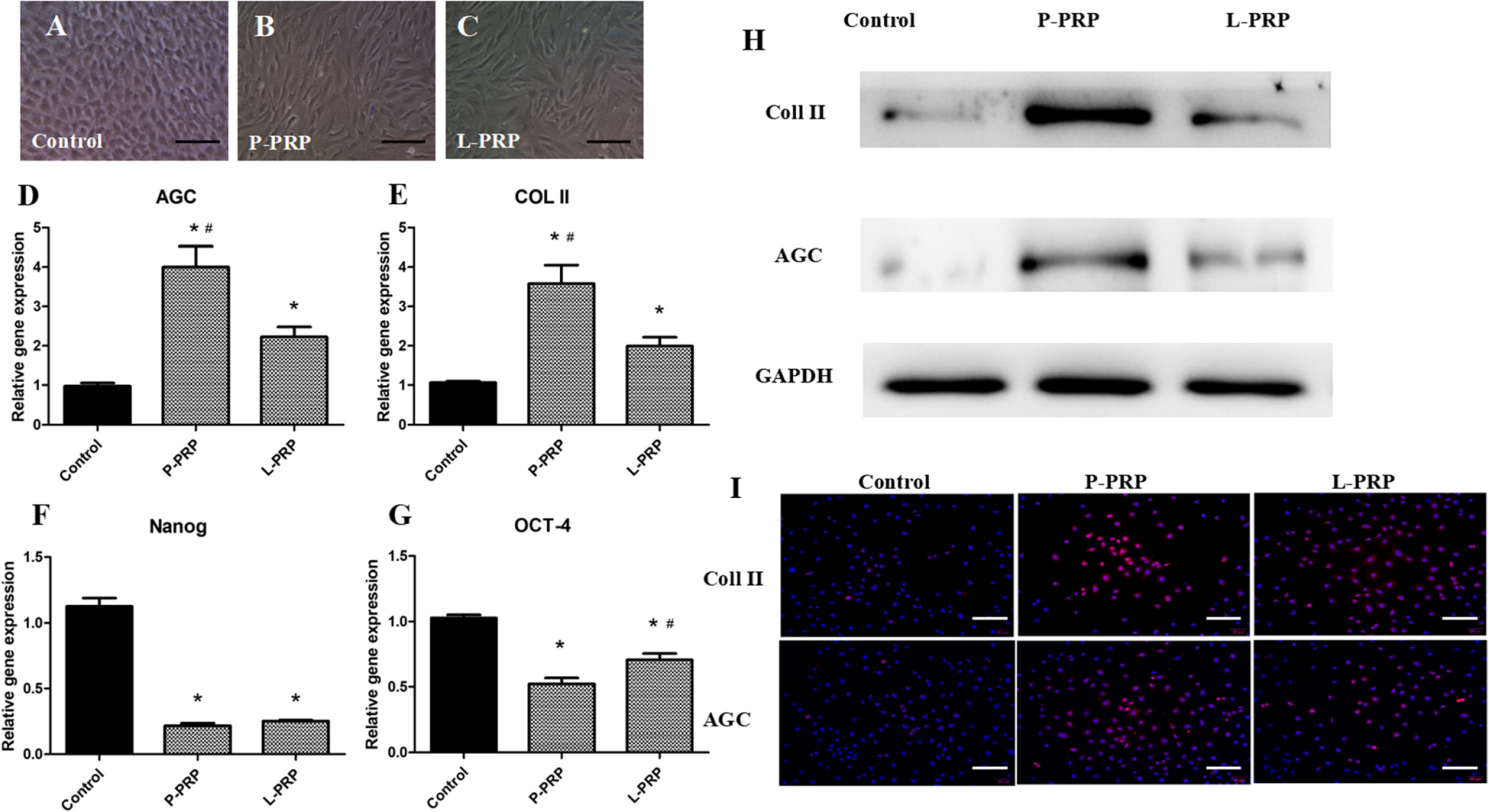
P-PRP and L-PRP induce NPSCs differentiation towards active NP cells. In the control group, cells were cobblestone-shaped, a typical feature of NPSCs (**a**). Either P-PRP or L-PRP treatment changed cell morphology into more elongated NP cells (**b**, **c**). P-PRP and L-PRP increased the expression of active NP cell genes, including Col II and AGC, when compared with the control group (**d**, **e**). Expression of stem cell marker genes, Nanog and Oct-4, was both reduced in P-PRP- and L-PRP-treated cells (**f**, **g**). Western blots validated increased Col II and AGC protein level induced by P-PRP and L-PRP compared with the control group (**h**). The active NP protein production was highest in P-PRP group. Immunostaining of Col II and AGC was positive in P-PRP and L-PRP group (*pink dots*), but negative in the control group (**i**). The highest protein staining intensity (*pink dots*) was observed in P-PRP group. Nuclei are stained *blue* with Hoechst 33,342. Significant differences (*P* < 0.05) are indicated by *asterisks*. Scale bars, 100 mm. *AGC* aggrecan, *Col II* collagen type II, *L-PRP* leukocyte-platelet-rich plasma, *P-PRP* pure platelet-rich plasma, *NP* nucleus pulposus, *NPSCs* nucleus pulposus-derived stem cells

NPSCs morphology in the controls maintained the shape of cobblestone-like cells (Fig. 6a). However, after the treatment of 10% L-PRP or 10% P-PRP, NPSCs proliferated faster and the shape changed from cobblestone-like to fibroblast-like shape (Fig. 6b, c). Two markers of active NP cells, including AGC and Col II increased significantly compared to the control group (Fig. 6d, e). P-PRP yielded the highest gene expression among the three groups. Furthermore, analysis of stem cell markers, including Nanog and OCT-4, indicated that both P-PRP and L-PRP decreased the stemness of NPSCs in vitro (Fig. 6f, g). Western blot analysis also validated these results (Fig. 6h). Immunostaining of the active NP cell protein, AGC, and Col II, was confirmed in high intensity after L-PRP or P-PRP treatment, while this was negligible in the control group. P-PRP treatment resulted in the highest protein production among the three groups (Fig. 6i).

### L-PRP induces higher levels of inflammatory responses in NPSCs from early degenerated discs

**Fig. 7:**
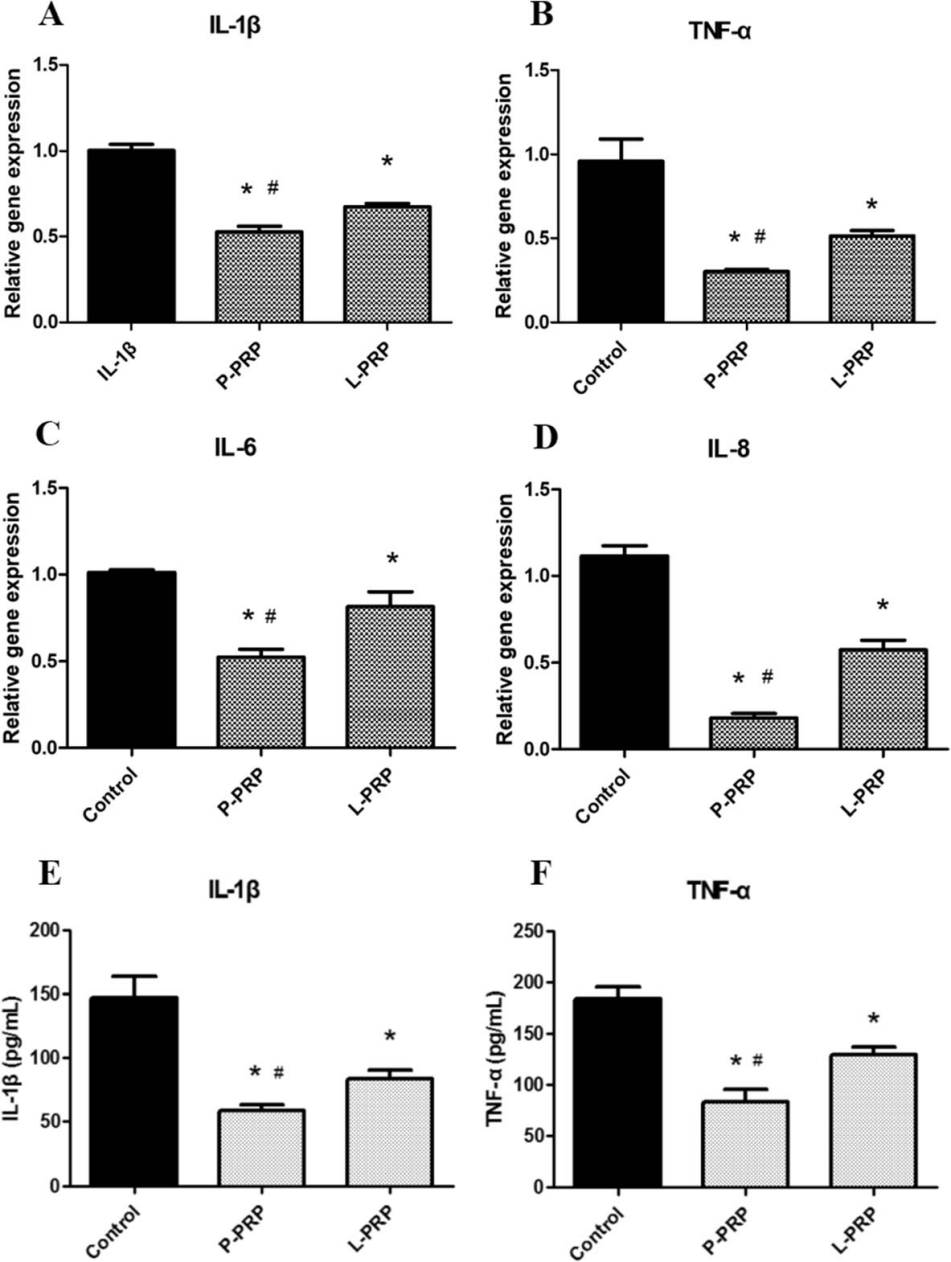
P-PRP induces less inflammatory responses than L-PRP. P-PRP and L-PRP both decreased the inflammatory gene expression of IL-1β and TNF-α (**a**-**d**). The inflammatory gene expression was significantly lower in P-PRP group. Moreover, the level of inflammatory cytokines (IL-1β, TNF-α) in the cell supernatants was consistent with the above gene expression data (**e**-**f**). *Asterisks* represent significant differences when compared with the respective control, and the *pound symbols* indicate significant differences between L-PRP and P-PRP (*P* < 0.05). *IL* interleukin, *L-PRP* leukocyte-platelet-rich plasma, *P-PRP* pure platelet-rich plasma, *TNF* tumor necrosis factor

To investigate the effects of L-PRP and P-PRP on the inflammatory responses, we first examined the expression levels of the inflammatory genes, IL-1β, TNF-α, IL-6, and IL-8, by qRT-PCR. Compared to the control group, P-PRP and L-PRP both decreased the expression of inflammatory genes (Fig. 7a-d). Also, we noticed that the inflammatory gene expression was significantly lower in the P-PRP group compared to that in the L-PRP group. Moreover, the levels of inflammatory cytokines (IL-1β, TNF-α) in the cell supernatants were consistent with the above gene expression data (Fig. 7e-f).

### P-PRP downregulates more extensive catabolic responses in NPSCs from early degenerated discs

**Fig. 8:**
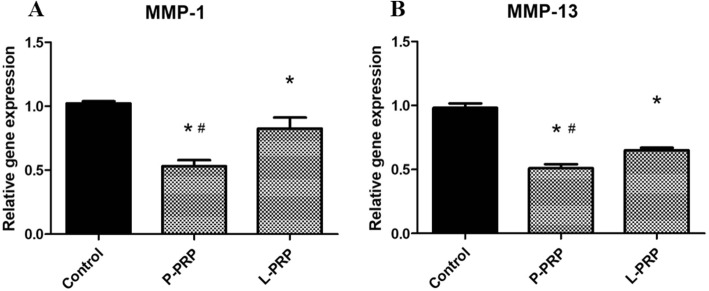
P-PRP downregulates more extensive catabolic responses. P-PRP and L-PRP both significantly downregulated the gene expression of MMPs (MMP-1, MMP-13) when compared with the control (**a, b**). P-PRP significantly downregulated the above catabolic genes compared with L-PRP. *Asterisks* represent significant differences when compared with the respective control, and the *pound symbols* indicate significant differences between L-PRP and P-PRP (*P* < 0.05). *L-PRP* leukocyte-platelet-rich plasma, *MMP* matrix metalloproteinase, *P-PRP* pure platelet-rich plasma

P-PRP and L-PRP both significantly downregulated the gene expression of MMPs (MMP-1, MMP-13) when compared with the control. P-PRP significantly downregulated the above catabolic genes compared with L-PRP (Fig. 8a, b).

## Discussion

PRP therapy has been widely applied in clinical repair and regeneration of different tissues and organs [16, 32, 33]. Many human clinical trials and basic scientific researches support the use of PRP not only for its autologous origin, but also for its activation of the tissue repair process [34–36]. PRP was proved promising in IDD by valuable clinical trials and animal studies [6, 37, 38]. However, inconsistency in composition of PRP prepared by different methods may have contributed to different results [39]. Leukocytes were suggested for their pro-inflammatory effect in PRP and were also proposed to be excluded in bone defects, chronic tendon injury, and osteoarthritis treatment [25]. Therefore, in this study, we determined whether the exclusion of leukocytes in PRP (P-PRP) was more valuable in repair and regeneration of early IDD.

In recent years, the endogenic stem cells from the NP tissues were isolated and characterized for its potential in self-renewal and tissue regeneration [23, 24]. In light of this, we tried to isolate stem cells from rabbit NP tissues in the early degenerated stage, which may potentially be a self-restorer when activated by P-PRP. In the early stage of IDD, the endogenic stem cells still existed, but the bioactivity was impaired. Compared to the colony-forming cells isolated from the healthy NP tissues, these cells from the early degenerated NP tissues formed less colonies and proliferated slower. A recent study showed similar results when comparing the stem cells isolated from human degenerated NP tissues with those from healthy ones [40]. The T2 signal intensity of MRI showed the worsening degeneration of the punctured discs with the extension of time in this study. Thus, active intervention for IDD in the early stage is particularly important for intervertebral disc regeneration.

To determine whether the isolated colony-forming cells expressed typical surface antigens of MSCs, we did not test the classic surface markers of MSCs for the lack of specific rabbit antigens by flow cytometry. Therefore, we used PCR to confirm the expression of these markers from gene level. The NP-derived colony-forming cells isolated from early degenerated discs (D-NPSCs) and healthy discs (H-NPSCs) both had strong expression of MSC-related markers (CD29, CD44, and CD166). Meanwhile, they had negligible expression of hematopoietic markers (CD4, CD8, and CD14), which seldom exist in MSCs. As controls, rabbit spleen cells, mostly lymphocytes and monocytes, were found to be strongly positive for both hematopoietic and MSC markers, while NP cells exhibited negligible expression of all the above genes.

The results of this study showed that the optimal proliferation rate of induced NPSCs was obtained at 10% P-PRP concentration. A dose-dependent effect was observed below 10% P-PRP concentration, which could be explained by more growth factors released from increasing PRP concentration. However, excessive supplementation of P-PRP in this study resulted in decreased proliferation rate, which was also confirmed in a recent study [25]. Thus, when designed in clinical use, PRP concentration and leukocyte exclusion should be carefully tested to achieve the optimal outcome.

Previous studies have not specifically investigated the differential effects of L-PRP and P-PRP on NPSCs. Our findings demonstrated that both P-PRP and L-PRP could induce differentiation of NPSCs into active NP cells. Analysis of stem cell markers, including OCT-4 and Nanog, indicated that both P-PRP and L-PRP decreased the stemness of NPSCs in vitro. The gene expression and protein production of NP-related ECM, including collagen II and aggrecan, was significantly upregulated. However, we noticed that P-PRP was superior in collagen II and aggrecan gene expression compared to L-PRP. Thus, when treating the early IDD, P-PRP injection may promote more synthesis and accumulation of NP-related ECM, which is important in the mechanical and biological reconstruction of the degenerated intervertebral discs.

Compared to the control group, P-PRP and L-PRP both decreased the inflammation and catabolic gene expression level (IL-1β, TNF-α, IL-6, IL-8, MMP-, and MMP-13) of the induced NPSCs. The results were similar to a study confirming that PRP was able to suppress cytokine-induced pro-inflammatory degrading enzymes and mediators in NP cells [41]. The inflammation and catabolic gene expression levels were significantly lower in P-PRP. These findings suggest that P-PRP may exert a lower inflammation influence on the treated degenerated intervertebral discs. Therefore, the preparation of PRP is a critical factor in IDD treatment, because different leukocyte levels can contribute to variances in therapeutic efficacy. A commonly used two-stage centrifugation method was applied in many commercial kits for clinical use. It was reported that PRP prepared by two commercial kits (GPS and ACP) contained active forms of MMPs, leading to the impairment of tissue regeneration [18]. Thus, a more advanced kit should be further designed to exclude leukocytes when applied in clinical treatment.

In this study, we did not investigate the effects of P-PRP on healthy NPSCs, but rather NPSCs from early degenerated discs. As suggested in IDD therapy based on biological strategies, early intervention is the key point for regeneration [9, 42, 43]. Previous studies have confirmed that the proliferation and differentiation potentials of NPSCs are worsening in the degeneration process [24, 40, 44]. In this study, we noticed that P-PRP and L-PRP both induced NPSCs from early degenerated discs into active NP cells by upregulating collagen II and aggrecan gene expression and protein synthesis. The results were consistent with some in vivo studies, which proved that PRP injection was helpful in restoring the ECM content, thus prohibiting the degeneration process [28, 45, 46]. However, we did not test the efficacy of P-PRP on the NPSCs from advanced degenerated intervertebral discs for those three reasons. First, in the late stage of IDD, NP structure collapses and can hardly be collected from rabbit models used in this study. Second, IDD is a progressive, chronic disease; over time, the degeneration worsens and ultimately becomes irreversible; in the late stage of IDD, the calcified endplate holds limited potential for vascularization and nutrient delivery for the starving cells [47, 48]. Third, IDD reconstruction by tissue engineering holds promise for late stage of IDD [49, 50]; the use of PRP is more beneficial when co-applied with more active seeding cells, but not the cells in degeneration or even senescence.

PRP has become a promising agent for treating surgical site infections for the antibacterial activity [21]. However, as for IDD, which mostly is related to the chronic low back pain, excluding leukocytes in PRP would be a more effective choice. The presence of pro-inflammatory cytokines and mediators is characterized as the prominent change in IDD [51]. The decreased inflammatory cytokines can prohibit the unwanted inflammatory-related pain and unnecessary ECM degradation. The injection agent for IDD should be safe, together with low pro-inflammatory effects. Leukocytes in PRP may only exacerbate IDD by prolonging the inflammatory process, leading to worsening pain experience in patients. Thus, P-PRP exhibits a superior choice compared to L-PRP in IDD treatment.

## Conclusions

In this study, we confirmed that the colony-forming cells (NPSCs) could be isolated from the NP tissues of early degenerated intervertebral discs. PRP, including P-PRP and L-PRP, could proliferate NPSCs and activate these cells toward active NP cells. Both two types of PRP could suppress the inflammatory levels of NPSCs cultured in vitro. However, P-PRP exerted a significantly lower catabolic and inflammatory effect on NPSCs. These results explain the exclusion of leukocytes from PRP may be a more effective choice for IDD in early stage.

## Abbreviations

ACD-A: Acid citrate dextrose solution A
AGC: aggrecan
bFGF: Basic fibroblast growth factor
CCK-8: Cell Counting Kit-8
Col II: Collagen type II
CTGF: Connective tissue growth factor
DMEM-LG: Dulbecco’s modified Eagle’s medium with low glucose
ECM: Extracellular matrix
EGF: Epidermal growth factor
IDD: Intervertebral disc degeneration
IL: Interleukin
L-PRP: Leukocyte-platelet-rich plasma
MMP: Matrix metalloproteinase
MRI: Magnetic resonance imaging
NP: Nucleus pulposus
NPSCs: Nucleus pulposus-derived stem cells
PCR: Polymerase chain reaction
PDGF: Platelet-derived growth factor
P-PRP: Pure platelet-rich plasma
PRP: platelet-rich plasma
qRT-PCR: quantitative real-time polymerase chain reaction
TGF-β: Transforming growth factor beta
TNF: Tumor necrosis factor
VEGF-A: Vascular endothelial growth factor-A
